# An Exploration Into the Use of a Chatbot for Patients With Inflammatory Bowel Diseases: Retrospective Cohort Study

**DOI:** 10.2196/15589

**Published:** 2020-05-26

**Authors:** Aria Zand, Arjun Sharma, Zack Stokes, Courtney Reynolds, Alberto Montilla, Jenny Sauk, Daniel Hommes

**Affiliations:** 1 Vatche and Tamar Manoukian Division of Digestive Diseases UCLA Center for Inflammatory Bowel Diseases David Geffen School of Medicine, University of California at Los Angeles Los Angeles, CA United States; 2 Department of Digestive Diseases Leiden University Medical Center Leiden Netherlands; 3 Cisco Systems Inc Collaboration Technology Group Dallas, TX United States

**Keywords:** chatbots, inflammatory bowel diseases, eHealth, artificial intelligence, telehealth, natural language processing

## Abstract

**Background:**

The emergence of chatbots in health care is fast approaching. Data on the feasibility of chatbots for chronic disease management are scarce.

**Objective:**

This study aimed to explore the feasibility of utilizing natural language processing (NLP) for the categorization of electronic dialog data of patients with inflammatory bowel diseases (IBD) for use in the development of a chatbot.

**Methods:**

Electronic dialog data collected between 2013 and 2018 from a care management platform (*UCLA eIBD*) at a tertiary referral center for IBD at the University of California, Los Angeles, were used. Part of the data was manually reviewed, and an algorithm for categorization was created. The algorithm categorized all relevant dialogs into a set number of categories using NLP. In addition, 3 independent physicians evaluated the appropriateness of the categorization.

**Results:**

A total of 16,453 lines of dialog were collected and analyzed. We categorized 8324 messages from 424 patients into seven categories. As there was an overlap in these categories, their frequencies were measured independently as symptoms (2033/6193, 32.83%), medications (2397/6193, 38.70%), appointments (1518/6193, 24.51%), laboratory investigations (2106/6193, 34.01%), finance or insurance (447/6193, 7.22%), communications (2161/6193, 34.89%), procedures (617/6193, 9.96%), and miscellaneous (624/6193, 10.08%). Furthermore, in 95.0% (285/300) of cases, there were minor or no differences in categorization between the algorithm and the three independent physicians.

**Conclusions:**

With increased adaptation of electronic health technologies, chatbots could have great potential in interacting with patients, collecting data, and increasing efficiency. Our categorization showcases the feasibility of using NLP in large amounts of electronic dialog for the development of a chatbot algorithm. Chatbots could allow for the monitoring of patients beyond consultations and potentially empower and educate patients and improve clinical outcomes.

## Introduction

### Background

Recent technological advances have allowed for artificial intelligence (AI) to successfully integrate itself into many aspects of daily life. Besides implementation in voice bots such as Amazon’s Alexa and Apple’s Siri, AI is also utilized to predict financial stock market changes and answer student questions in educational settings [1]. In health care, AI is expected to disrupt the role of physicians as well; however, experts predict that AI will support the intelligence and knowledge base of physicians rather than replace them entirely [2]. For instance, AI can utilize deep-learning algorithms, which function like the neural networks of the brain and distinguish patterns, to recognize certain types of brain tumors, vascular conditions, or pneumonia on imaging scans and prioritize these cases in the workflow of a radiologist [2,3]. In addition, AI can be used to quickly review patient scans and rule out certain diagnoses, thereby increasing the efficiency and accuracy of a radiologist [2].

Another significant way AI can augment health care delivery is through medical chatbots. A chatbot, or chatterbot, attempts to simulate a natural conversation with a human user [4]. Medical chatbots are already being implemented into regular practice: the Insomnobot-3000 helps insomniacs get through the night, and the Endurance bot acts as a companion for dementia patients [5]. In addition, there are significant efforts toward the development of diagnostic chatbots. Some popular ones include Your.MD, Buoy Health, Sensely, Infermedica, and Florence (Table 1) [6].

Although there are limited data on these general medical chatbots in clinical practice, some independent bodies have provided preliminary and positive results in tests with more specific medical chatbots [7,8].

Most chatbots utilize natural language processing (NLP), which can be simply defined as the use of computers for analyzing human language [9]. One application of NLP relies on human identification of key elements within an event or situation that might constitute a useful summary of a given document or dataset [10]. Recently, there have been growing trends toward the use of electronic health records (EHRs). Multiple studies have attempted to use NLP to extract useful information from EHRs. In one study, researchers used NLP to identify patients with ulcerative colitis and Crohn disease from EHR data collected from Massachusetts General Hospital and Brigham and Women’s Hospital [11]. The study developed an algorithm that partly relied on recognizing keywords associated with ulcerative colitis or Crohn disease to analyze the narrative texts and was verified via comparison to a physician’s review and classification of the same narrative texts [11]. Ultimately, the study determined that NLP of patient narrative texts provided a more accurate means of identifying patients who had ulcerative colitis and Crohn disease than previous models that had relied on reviewing billing codes [11].

In another study by the University of Alabama, researchers developed an algorithm that analyzed the EHRs of patients collected over 3 years and organized the EHRs into pathology clusters based on key terms [12]. This team also concluded that electronic text mining of health records, or NLP, is an effective method for analyzing large health care datasets [12]. More recent studies have even attempted to use NLP models to study the semantics and sentence flows found in clinical narrative data [13,14]. The literature shows that it is common to perform exploratory analysis on natural language data to understand the topics and vocabulary of a specific domain in health care [9-14]. This exploration is often done by grouping keywords and categorizing topics or using open-source technology such as clinical Text Analysis and Knowledge Extraction [13]. A deep initial understanding facilitates the creation and comparison of more complex, health care-focused NLP models. However, it is worth noting that certain aspects of patient consultations in clinical settings, such as electronic record style, patient behavior, and physician experience, can vary from clinic to clinic [9,14]. This variability found within patient data puts limits on what NLP can do without a large and diverse sample.

In addition, despite the extensive literature on the topic, there seems to be a lack of research into the use of NLP to analyze raw consultation dialog data of patients with specific chronic conditions such as inflammatory bowel diseases (IBD). The organization of the patient with IBD to health care provider (HCP) dialog is likely to be distinct from a general patient population due to the complex nature of the disease. Understanding how these dialogs can be organized is an important first step in assessing the feasibility of a chatbot for this population.

Chatbots that utilize NLP can help to improve the way health care is delivered in multiple ways. For one, they improve accessibility to health care for patients outside of clinics and hospitals. From kids to the elderly, patients often need care outside of inpatient consultations; lack of such support is associated with inefficiency, high health care costs, and burdened HCPs [15]. With a chatbot, these patients would have immediate and autonomous support at home.

**Table 1:** Overview of current medical chatbots.

Name	Disease area	Objective	What does it do
Your.MD (UK^a^)	General	Provide reliable information for common symptoms, recommends relevant resources	Safely advises patients based on symptoms described in an app-based messaging system
Endurance (Russia)	Dementia	Act as a companion for patients with short-term memory loss and help to identify signs of worsening patient condition	It works via voice recognition to ask questions and react to answers. It can speak on a variety of topics and pull interesting news from Google
Insomnobot-3000 (US^b^)	Insomnia	Acts as a companion for insomniacs when they are awake at night.	Has conversations with patients via text
Pharmabot (Philippines)	Pediatrics	Designed to help pediatric patients get appropriate generic medicine for certain ailments	The system works in a software application that sets particular guidelines for interaction with the chatbot
Text-based healthcare chatbots on Mobile Coach (Switzerland)	Childhood obesity	Provide a peer character for obese teenagers and keep them engaged. In addition, sought to show the benefit of text-based chatbot interventions in health care	Works in a text channel within an app interface. Also, has predefined answer options for more efficient chat interactions
Molly by Sensely (US)	General	Diagnose patients with common ailments appropriately based on symptoms	Advises patients based on symptoms described in an app-based messaging system
Buoy Health (US)	General	Diagnose patients accurately based on symptoms. Harvard team developed the algorithm for this bot using 18,000 medical papers for data	Program asks a series of questions—for which there are predefined choices to choose from—to appropriately advise patient. Found on a Web-based software
Symptomate by Infermedica (Poland)	General	Attempt to increase health care provider efficiency, reduce costs, and improve patient flow by acting as a general symptom checker	Online software that collects and analyzes symptom data via predefined questions with answers to provide appropriate response
Florence (Germany)	General	Acts as a *personal nurse* that can remind patients to take prescriptions and keep track of user’s health (weight, mood, etc)	Advises patients based on symptoms described in an app via Facebook messenger
Ada (international)	General	Help patients actively manage health based on common symptoms	Ada poses simple and relevant questions to patients and then compares their symptoms with thousands of similar cases to help provide possible explanations
Holly by Nimblr (US)	N/A^c^	Helps patients schedule and reschedule appointments to help prevent no shows or cancellations and improve patient experience	Interacts with patients via text and Amazon’s Alexa to update electronic health records
Woebot (US)	Psychiatry	Make mental health care more accessible to people around the world	Uses methods from cognitive behavioral therapy to help patients think through situations. It also includes intelligent mood tracking

^a^UK: United Kingdom.

^b^US: United States.

^c^N/A: not applicable.

### Objectives

The primary objective of this study was to accurately categorize large datasets of electronic messages between patients with IBD and HCPs using natural language processing (NLP) to assess the feasibility of developing a medical chatbot for patients with IBD.

## Methods

### Design and Population

In this study, we aimed to assess the feasibility of utilizing NLP on historical electronic messaging data of patients with IBD for use in the development of a medical chatbot. As IBD is a chronic illness characterized by severe and recurring abdominal pain and diarrhea, patients require frequent contact with their physicians and care team to monitor these alternating disease states and potential relapses [16]. There is great potential here for a chatbot as patients need frequent monitoring beyond regular consultations, which is often troublesome due to the complex nature of the disease and a busy care team.

**Figure 1:**
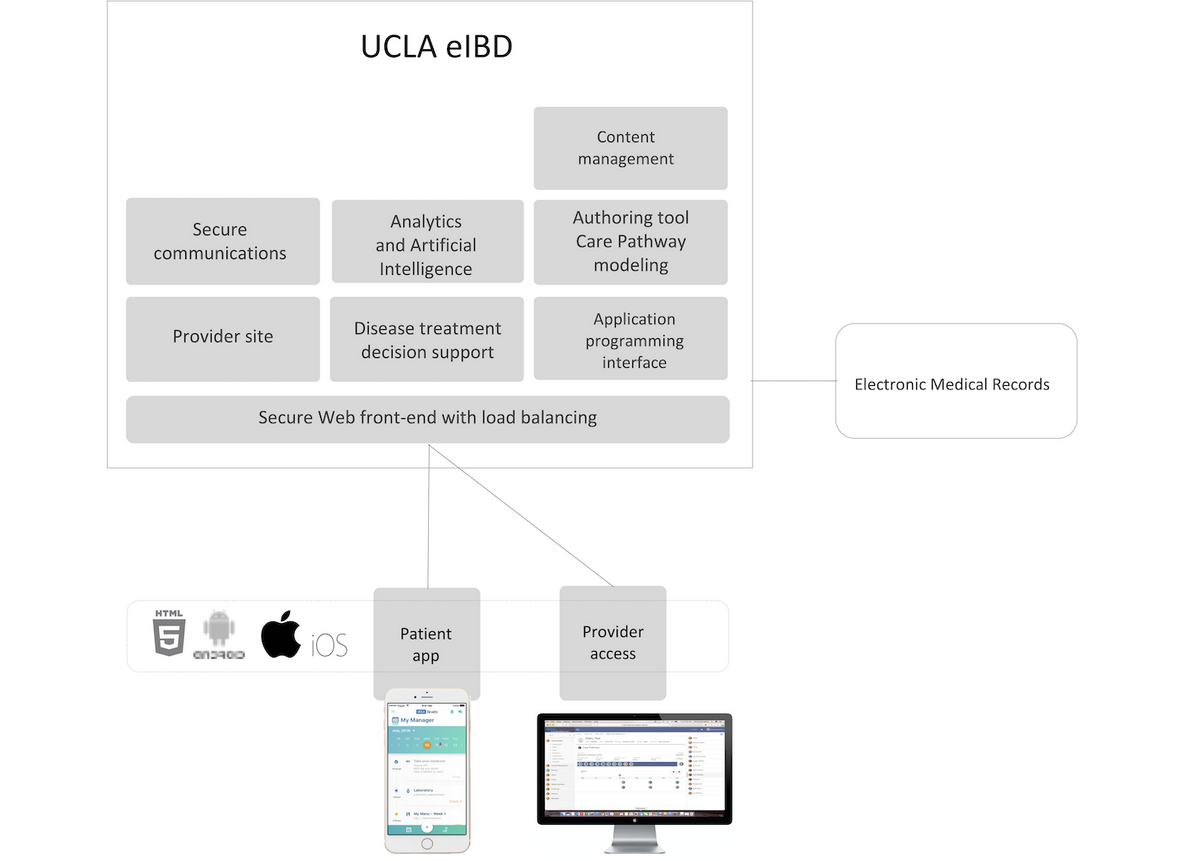
Overview of UCLA eIBD platform. AI: artificial intelligence; API: application programming interface.

Patients enrolled in the University of California, Los Angeles (UCLA) Center for IBD electronic care management platform (UCLA eIBD) were retrospectively assessed. The UCLA eIBD platform is a care management software as a service with a Web-based platform for providers that includes treatment decision support, business intelligence, messaging functionality, and performance improvement tools. On the patient’s side, there is a mobile app that includes care management insight, educational modules, surveys, and messaging (Figure 1) [16]. Retrospective dialog data between patients and their care team from 2013 until 2018 was extracted and the feasibility of applying NLP categorization algorithms was assessed.

All patients gave informed consent to participate. This study was approved by the Institutional Review Board (IRB) at UCLA with IRB protocol number 17-001208.

### Data Collection and Anonymization

The dialogs were extracted from the UCLA eIBD database. The data consisted of the following: (1) a unique identifier, (2) first name, (3) last name, (4) date and time of message, (5) direction of message (HCP to patient or vice versa), (6) message content, (7) potential attachments, (8) HCP classification (urgent and nonurgent), (9) HCP action (responded yes or no), and (10) HCP response message content (Multimedia Appendix 1). The data were anonymized by removing the first and last names; for identification, we made use of the unique identifier in our analysis.

### Categorization Method: Use of Natural Language Processing

**Figure 2:**
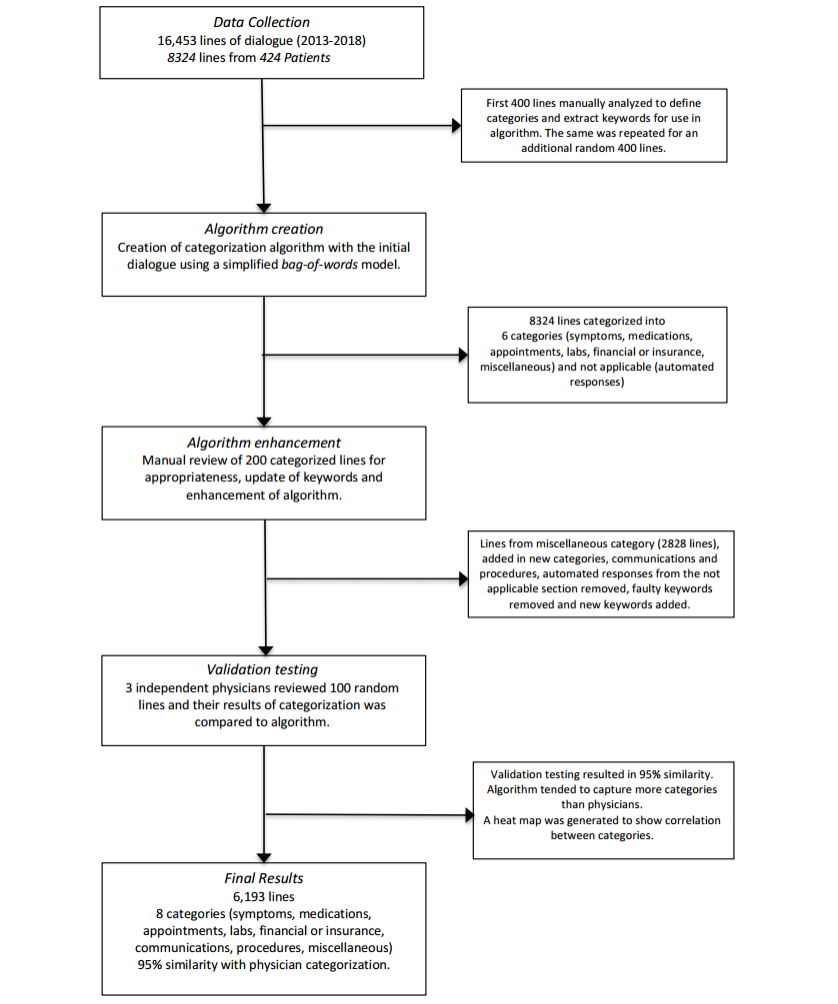
Flowchart of inclusion and categorization. N/A: not applicable.

Once the patient to HCP dialogs were stored in a Microsoft Excel sheet, the first 400 lines within the sheet were manually analyzed to identify relevant categories for use in our NLP algorithm. To clarify that the first 400 lines were representative, an additional 400 lines were randomly generated and manually reviewed as well (by AS and ZS). The analysis consisted of reading over each line to find an intent; if a particular intent was seen to occur frequently in these first lines, it was noted as a relevant category. The rationale behind using only categories observed in the sample was to make sure that the categories coded for were relevant to what the patient sample was discussing with their HCPs. Furthermore, 2 IBD gastroenterologists reviewed the categories found from the sample and reaffirmed that each category was representative of the IBD patient conversations they had encountered through electronic channels such as email. The same first 400 lines were then used to identify which keywords could assign a given dialog to a certain category (Multimedia Appendix 2). If a term appeared roughly 10 or more times in a given category, it was noted as a potential keyword; 2 physicians then reviewed and approved our list terms. Using these keywords, we employed a simplified, rule-based bag-of-words model to assign each line of dialog to the appropriate categories (Figure 2). The bag-of-words model essentially allows one to extract particular features of a text, that is, keywords, and score them with relevant numbers for modeling, or in our case, categorization [17]. To be certain, each line was converted into a standard bag-of-words with a score for each word in the form of a count of the number of times it appears within the line. With stop words removed, we extract the score of each keyword from all lines and assign to each line all categories for which any one keyword has a positive score.

### Enhancement and Correlation Assessment

On the basis of the preliminary results, the keywords of our initial categorization algorithm were refined, and new categories were created if necessary. If the categorization was not logical, we analyzed which keywords in the model miscategorized the dialog and made the necessary improvements. In addition, any uncategorized lines of dialogs were assigned a category, and their keywords were identified. The categorization algorithm was updated with the new, physician-approved keywords extracted from the uncategorized lines of dialog and the improvements of the existing categorization.

Once the code was refined to capture all the lines of dialog, a heat map was generated to showcase the overlap in categories, which refers to one line of dialog from a patient falling into two categories. It is worth noting that more than two categories could overlap, but there was no way to represent the higher levels of overlap in a relevant and concise diagram such as a heat map. The goal was to paint a picture of what types of questions or concerns popped up together, which is instrumental in the actual development of a chatbot and creation of multicategory scenarios.

### Validation of Accuracy

The accuracy of our categorization algorithm was tested by having 3 independent physicians from the UCLA Division of Digestive Diseases (AZ, CR, and DH) evaluate the appropriateness of the categorization. Each physician was assigned to categorize 100 randomly collected lines of dialog using the defined corresponding category number. In addition, the physicians categorized each line in the same style as the algorithm: numerical order with no spaces.

Once each of the doctors had finished categorizing the lines, the results were compared with the algorithm’s categorization. We showcased the extent to which the algorithm and the doctors agreed or disagreed. To do this, the number of underclassifications and overclassifications the categorization algorithm made relative to the doctors’ categories was calculated. For instance, if the algorithm missed a category that the doctor had, it would be counted as an underclassification of 1; if the category code had an extra category compared with the doctor, it would be counted as an overclassification of 1. We then created a bar chart plot based on this data. In addition, to understand the practicality of treating the doctors’ assessments as ground truth, we computed the level of agreement between the three raters using Krippendorf alpha. This is a standard estimate of inter-rater reliability across ratings on a nominal scale.

To calculate a metric for the accuracy of the algorithm itself, we opted to use a nonstandard method of computing the success of the classification algorithm in an attempt to incorporate expert knowledge about the severity of misclassifications. As standard reliability measures such as Krippendorf alpha treat all disagreements between the raters and the algorithm with equal weight, we would not get a realistic view of the algorithm’s strength across the spectrum of categories by following this approach. This was also done in an attempt to avoid aggregating our multiclass labels from the raters as doing so would put us at risk of destroying the variability in the ratings and inflating performance.

### Software

Excel 2010 and R studio programming tool (R 3.4.0) were used for our analysis and algorithm creation (Multimedia Appendix 3).

## Results

### Data and Population Characteristics

Our sample consisted of 424 patients, 3 physicians, 3 nurses, and 2 administrative assistants with 16,453 lines of electronic dialog. Of the dialogs, 8324 lines were sent by 424 patients to their HCP (patient to HCP). Our analyzed patient cohort is 51.9% (220/424) female, 50.7% (215/424) have Crohn disease, and 46.9% (199/424) have ulcerative colitis with a mean disease duration of 13.4 (SD 10.4) years. The majority of the population is of the white (284/424, 67.0%) race and not of Hispanic or Latino ethnicity (386/424, 91.0%). Furthermore, most of the patients are employed (283/424, 66.7%) and have been enrolled in the care program for a mean of 4.6 (SD 1.3) years (Table 2).

**Table 2:** Characteristics of the inclusion cohort (N=424).

Variable		Values
Age (years), mean (SD)		42 (14)
**Gender, n (%)**		
	Female	220 (51.9)
	Male	204 (48.1)
**Disease type, n (%)**		
	Crohn disease	215 (50.7)
	Ulcerative colitis	199 (46.9)
	Indeterminate colitis	10 (2.4)
Disease duration (years), mean (SD)		13.4 (10.4)
**Race, n (%)**		
	White	284 (67.0)
	Unknown	97 (22.9)
	Asian	26 (6.1)
	Black or African American	12 (2.8)
	American Indian or Alaska Native	4 (0.9)
	Native Hawaiian	1 (0.2)
**Ethnicity, n (%)**		
	Not Hispanic or Latino	386 (91.0)
	Hispanic or Latino	29 (6.8)
	Unknown	9 (2.1)
**Employment, n (%)**		
	Employed	283 (66.7)
	Unemployed or unknown	141 (33.2)
Duration in program (years), mean (SD)		4.6 (1.3)

### Algorithm Development and Initial Results

In our manual run-through of the first 400 out of the 8324 lines of dialog, we categorized them in six newly created and distinct categories: (1) medications, (2) symptoms, (3) appointments, (4) laboratory investigations, (5) finance/insurance, and (6) miscellaneous (lines that did not fall into any of the other categories). When the additional randomly generated 400 lines were reviewed for clarification, the same five relevant categories were found. At this point, we also kept a not applicable (N/A) section for automated responses produced by the mobile app itself that were in the dataset. For instance, “Patient has indicated there are no changes to medications.”

We identified what keywords were relevant to each of the categories (Multimedia Appendix 2). A categorization algorithm (bags-of-words model) was created based on the keywords extracted from the dialogs in the categories and applied to categorize the remaining lines of dialog.

Out of the 8324 lines of dialogs, the algorithm initially returned symptoms (1781/8324, 21.40% lines), medications (2114/8324, 25.40% lines), appointments (1781/8324, 21.40% lines), laboratory investigations (1648/8324, 19.80% lines), finance or insurance (358/8324, 4.30% lines), miscellaneous (2830/8324, 34.00% lines), and N/A (666/8324, 8.00% lines).

### Enhancement of Natural Language Processing Categorization Algorithm

The miscellaneous section (2828/8317, 34.00% lines) was manually reviewed for 200 lines. The miscellaneous section was essentially randomly generated in that it was not organized by any dialog identifier, such as medical record number or patient name; it was simply the arbitrarily leftover dialogs from our initial run of the algorithm. As the dialogs here were short and not dominated by any one patient, we found it appropriate to review the first 200 lines as an accurate representation of the larger section. On review, two additional categories were identified within it: communications and procedures. In addition, the miscellaneous category was analyzed for keywords that would improve the scope of our initial categories. For instance, there were some medications we missed in our first test, such as Tylenol, that we were able to find upon review of the miscellaneous section and add as a keyword for medications. Furthermore, we removed keywords from the algorithm that were too general and inflated certain categories, such as the keyword *take* for the medications category.

Finally, the categorization algorithm was enhanced to remove dialog that only contained generic greetings, such as *Thank you* or *Hello*, and the automated responses from the N/A section from the dataset so that they did not affect the final counts. After this enhancement, 2131 lines were excluded and 6193 lines of dialog were left for categorization.

### Final Natural Language Processing Categorization Results

**Figure 3:**
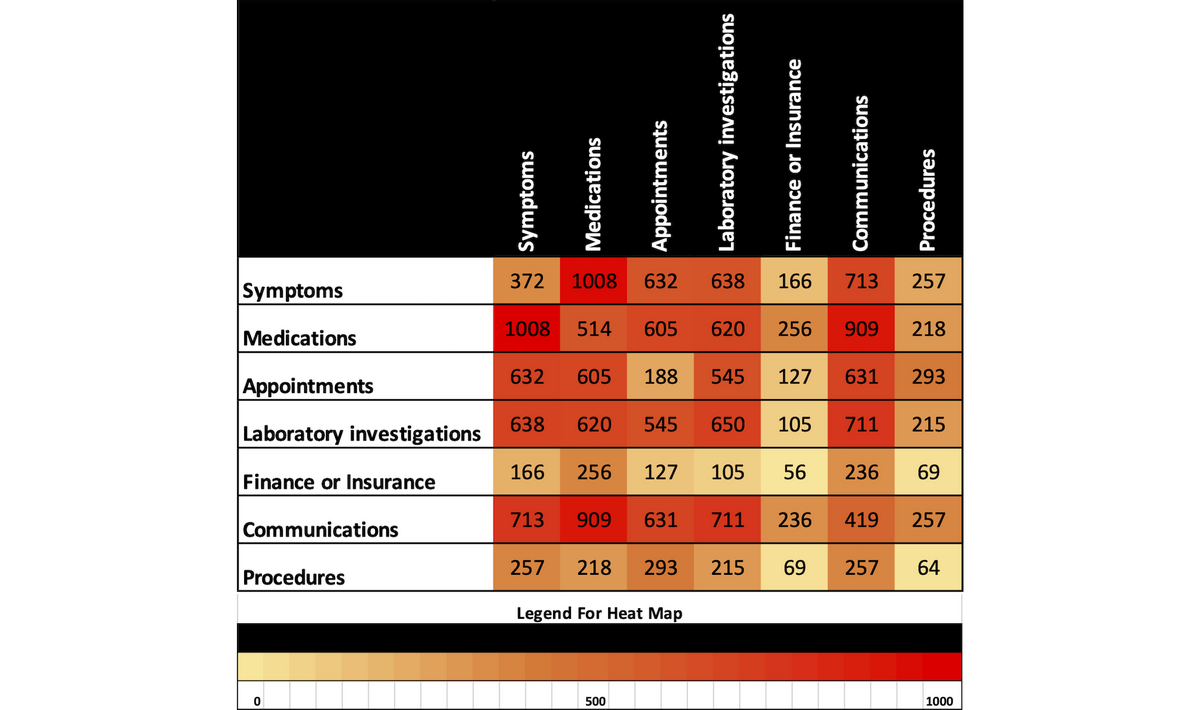
Heat map of category overlaps in dialog. This map shows the frequency of category overlap in pairs and how often the categories occurred by themselves out of the 6193 dialogs. Note: across the diagonal, the map is a mirror of itself.

These refinements ultimately led to the algorithm yielding 32.83% (2033/6193) of the dialog relating to symptoms, 38.70% (2397/6193) to medications, 24.51% (1518/6193) to appointments, 34.01% (2106/6193) to laboratory investigations, 7.22% (447/6193) to finance or insurance, 34.89% (2161/6193) to communications, 9.96% (617/6193) to procedures, and 10.08% (624/6193) being miscellaneous (Table 3). The frequency of this overlap was measured for each possible pair combination of the categories and is displayed in a heat map (Figure 3). For instance, medications and symptoms appeared more together than they did on their own, as did communications and symptoms. Similarly, procedures and finance were very rarely brought up on their own (Figure 3).

**Table 3:** Final categorization results (N=6193).

Category	Percentage of total sample^a^, n (%)
Symptoms	2033 (32.83)
Medications	2397 (38.70)
Appointments	1518 (24.51)
Laboratory investigations	2106 (34.01)
Finance or insurance	447 (7.22)
Communications	2161 (34.89)
Procedures	617 (9.96)
Miscellaneous	624 (10.08)

^a^These percentages represent how frequently these categories occur in the sample of dialogs. As the categories mostly overlap in the dialogs, the percentages do not add up to 100%.

### Validation of Natural Language Processing Accuracy

Three independent raters (AZ, DH, and CR) categorized 100 random lines of dialog, and their categorization was compared with our algorithms. The raters categorized in the exact style of the algorithm, so if the categories were symptoms, appointments, and medications, they would write *123*. Applying Krippendorf alpha to these assessment ratings, we get an estimate of .61, indicating that there was moderate-to-high agreement between the doctors.

In our underclassification and overclassification representation of the chatbot’s accuracy, we found that most of the errors were pooled at one difference, suggesting that the code and the doctors had a high level of agreement on most of the dialogs. Furthermore, the graph we constructed shows that the category code tended to over classify rather than under classify the subjects of the dialogs (Table 4). As one can see from the table, there is a significant drop in the instances of two or more underclassifications, with four to five missed categories having a frequency of 0 (Table 4). When we accounted for the 1 to 2 overclassifcation differences and the one category underclassification differences as minor, we found that 285 of the 300 tests had the program and physicians reasonably agreeing on categories. This meant that our code showed minor to no differences in 95% (285/300) of cases.

**Table 4:** Accuracy test results.

Number of categories added or missed by the algorithm in a given line	Instances in sample for overclassification, n	Instances in sample for underclassification
1	71	47
2	29	5
3	5	1
4	3	0
5	1	0

## Discussion

### Principal Findings

We were successful in categorizing large amounts of electronic messages between patients and providers into a reasonable number of categories (<10). Roughly 90.00% (5574/6193) of dialogs that came from patients fell into only seven categories, which shows potential for developing a chatbot with an NLP algorithm that can handle most IBD patient’s questions and concerns. In addition, our heat map gave us insight into how these categories correlate with each other in the dialogs. In terms of chatbot development, this map allows a developer to be aware of what categories or topics tend to appear together in patient with IBD to HCP dialogs. This insight would allow the developer to better prepare the chatbot's NLP algorithm to identify topic transitions in a patient conversation and respond appropriately. In addition, our accuracy test supported the reliability of this result. Most of the differences recorded in our test (100/162, 61.0%) were simply due to code over classifying with one or two categories, but it rarely missed the primary intent (Table 4). Even when it did miss a category relative to the physician, the program was not necessarily incorrect upon review. For instance, one of the dialogs in the accuracy sample had a patient describing their symptoms or medications and subtly mentioning their laboratory investigations as their *previous averages*. Although the doctors recognized this and appropriately categorized the line as symptoms, medications, and laboratory investigations, the algorithm categorized it as symptoms and medications only, as averages was not a keyword we had programmed for laboratory investigations. Despite this, the program correctly identified the primary intent of the dialog, which is why we considered these types of differences minor in measuring the accuracy of our program.

### Limitations

One limitation of this study is that our patient sample is fairly homogenous, consisting of mostly young (mean age 42 years) and white patients, which limits the generalizability of our results to other populations. In addition, most of the patients in the study are employed, which could have potentially changed the types of questions or concerns they expressed and the overall category distribution relative to other patient populations. It is also worth noting that we used the expert opinions of 2 IBD gastroenterologists to support the validity of the categories chosen and the selected keywords. This may affect the reproducibility of our results.

### Comparisons With Prior Work

The next step from collecting data to developing a chatbot is to use machine learning methods to model the relationship between questions and responses [18]. Many chatbot knowledge bases (the database from which a chatbot draws its responses from) are hand constructed, which is time consuming and reduces the algorithm’s versatility [19]. For instance, Artificial Linguistic Internet Computer Entity and *ELIZA*, two classic chatbots, utilize hand-constructed databases to generate a response that matches a given human input [20]. As an alternative, some developers have attempted to extract high-quality dialog data from online discussion forums to efficiently create a knowledge base for specific domain chatbots [19]. The purpose of collecting these dialog datasets is to give the chatbot a training ground to learn how to accurately respond to a specific domain of human input responses with minimal human fine tuning, or simply put: machine learning [18,21]. This machine learning approach also allows for the chatbot to continue learning through its interactions and improve its accuracy. Microsoft’s Xiaoice chatbot has successfully applied this model and has already amassed a following of about 660 million online users [22]. When assessing the appropriateness of our data for actual chatbot development, our code could be distributed and tested in other centers with the same historical data without requiring much customization and would eliminate the need for hand-constructed databases.

### Conclusions

Looking at the global trends of technology in health care, usage of smartphones and electronic health apps is on the rise [2,4,6]. Patient-provider communication through electronic messaging apps is becoming the standard. In our population, 25.0% (1518/6193) of messages were related to appointments. A chatbot could effectively automate requests regarding booking and cancellations or even play an instrumental part of triage, following the same guidelines as nurses, saving the provider team valuable time that could be redistributed to better patient care. The benefit is that a chatbot is available at all times, can handle tremendous amounts of conversation, and has no wait times.

Through the UCLA eIBD platform, we have already created a high-quality knowledge base of human dialogs that can be used to train an IBD chatbot using NLP. We showcased that it is feasible to categorize large amounts of electronic messaging data in one of the most complex chronic conditions into a reasonable number of categories. Given the feasibility of this categorization and the potential benefits of a chatbot, the next step would be to develop a chatbot and test it in a patient population with IBD. Further studies are required to showcase the effect on patients, providers, and costs and potential extrapolation to other chronic conditions.

## Appendix

Multimedia Appendix 1 Section 1, Table of dialog data content.

Multimedia Appendix 2 Section 2, Table of keywords for categories.

Multimedia Appendix 3 Section 3, Copy of natural language processing algorithm code.

## Abbreviations

AI: artificial intelligence
EHR: electronic health record
HCP: health care provider
IBD: inflammatory bowel diseases
IRB: Institutional Review Board
N/A: not applicable
NLP: natural language processing
UCLA: University of California, Los Angeles
