# Supplementary material for: An Exploration Into the Use of a Chatbot for Patients With Inflammatory Bowel Diseases: Retrospective Cohort Study
Source: J Med Internet Res. 2020 May 26;22(5):e15589. doi: 10.2196/15589 (PMC7284401; doi:10.2196/15589)
Supplement: Multimedia Appendix 1 [file jmir_v22i5e15589_app1.docx]

**Supplementary Table 1. Dialogue data content**

| Unique Identifier | Report Messages Received/Sent by |
| --- | --- |
| Report Messages Content | Report Messages Nurse Note Content |
| First name |  |
| Last name |  |
| Report Messages Patient Alert |  |
| Report Messages Date & Time |  |
| Report Messages Nurse Alert |  |
