# Supplementary material for: An Exploration Into the Use of a Chatbot for Patients With Inflammatory Bowel Diseases: Retrospective Cohort Study
Source: J Med Internet Res. 2020 May 26;22(5):e15589. doi: 10.2196/15589 (PMC7284401; doi:10.2196/15589)
Supplement: Multimedia Appendix 2 [file jmir_v22i5e15589_app2.docx]

**Supplementary Table 2. Keywords for Categories**

| **Category of Dialogue** | **Description** | **Keywords** |
| --- | --- | --- |
| Symptoms | Patient describing characteristics of ailment/problem they are having. | "I'm noticing", "be concerned", "diagnose", "I have been" ,"breaking", "ability", "I have a", "figure out", "pale", "I haven't had", "nausea", "weight", "anemia", "restroom", "bathroom", "stomach pain", "weaken", "sore", "serious pain", "infection", "bloated", "kidney", "itch", "tendon", "sensation", "bowel movement", "sick", "BM", "discomfort", "hurts", "my disease", "pooping", "GI track", "strokes", "spots", "sleep", "ache", "recovering", "BLEEDING", "reaction", "Crohn", "effect", "affect", "symptom", "feel", "problem", "fever", "cramp", "I was experiencing", "I've been", "I've had", "rash", "inflammation", "bleeding", "depression", "anxiety", "stool", "Stool", "depressed", "having pain", "abdominal pain", "medicine" |
| Medications | Any mention of or changes to a patients medications. | "meds", "prescription", "drug", "treatment", "infusion", "injection", "Vaccine", "taking", "prescribe", "prescription", "refill", "take the", "tabs", "daily", "tablet", "pill", "vaccinate", "miralax", "Miralax", "laxative", "Antibiotic", "antibiotic", "steroids", "supplement", "My medication", "my medication", "vaccine", "shot", "flu shot", "oral", "Flu shot", "the medication", "Walgreens", "walgreens", "CVS", "cvs", "pharmacy", "Pharmacy", "over the counter", "mg", "miligrams", "dose", "dosage", "pro biotic", "probiotic", "Probiotic", "Entyvio", "entyvio", "6MP", "6mp".... (Additionally, listed out about 50 different medications used by the UCLA IBD Center as keywords.) |
| Appointments | Patients trying to schedule appointments with provider. | "scheduling", "apt"," appointment", "see me", "see her", "see him", "see Dr", "see the", "seeing", "appt", "I can make", "schedule", "come in", "be there", "head over", "followup", "visit", "SEE OR", "meet" |
| Labs | Any question or concerns (troubleshooting, results, etc.) the patient may have. | "lab", "Lab", "results", "blood test", "CBC", "blood panel", "draw", "result", "blood work", "Quest", "quest diagnostic", "sample", "drew blood", "tests", "CRP", "test for", "bloods", "more blood", "this test", "my blood", "Vitamin D", "vitamin D", "Vitamin d", "iron", "glucose" |
| Finance/Insurance | Patient discussing any questions or concerns related to monetary issues. | "insurance", "cost", "careplan", "expensive", "money", "health plan", "$", "paystub", "Blue Shield", "financial", "funds", "PPO", "HMO", "Tricare", "tricare", "medical bills", "pricing", "Remistart", "remistart", "Co-Pay", "co-pay", "Healthcare" |
| Communications | The patient trying to get ahold of providers or leaving their contact information. | "E-mail", "email", [@gmail.com](http://@gmail.com), "[altour.com](http://altour.com/)", “[@mednet.ucla.edu](http://@mednet.ucla.edu)", "phone", "number", "my cell", "fax", "message", "Email", "error", "call", "get a hold of", "contact", "speak", "mail", "Zip code", "located", "location", "address" |
| Procedures | Patient discussing any questions or concerns related to procedures. | "colonoscopy", "procedure", "scopy", "MRI", "PT scan", "Petscan", "CT", "CAT", "x-ray",  "X-ray", "surgery", "biopsy", "biop", "TB test", "tuberculosis" |
