# Supplementary material for: An Exploration Into the Use of a Chatbot for Patients With Inflammatory Bowel Diseases: Retrospective Cohort Study
Source: J Med Internet Res. 2020 May 26;22(5):e15589. doi: 10.2196/15589 (PMC7284401; doi:10.2196/15589)
Supplement: Multimedia Appendix 3 [file jmir_v22i5e15589_app3.docx]

**Supplementary Table 3. Algorithm Code**

| setwd("I:/IBDcenter/`STUDIES/Chat-Bot") Chat = read.csv("Chat.csv",header=TRUE, stringsAsFactors = FALSE) Messages = Chat[,c(5,6,7)] Messages[,2] = 0 MessagesHP = subset(Messages, grepl(levels(factor(Messages[,1]))[1], Messages[,1])) MessagesPH = subset(Messages, grepl(levels(factor(Messages[,1]))[2], Messages[,1]))  ########Categorization CODE################################################################################################################################ one = c("I'm noticing","be concerned","diagnose","I have been","breaking","ability","I have a","figure out","pale","I haven't had","nausea","weight","anemia","restroom","bathroom","stomach pain","weaken","sore","serious pain","infection","bloated","kidney","itch","tendon","sensation","bowel movement","sick","BM","discomfort","hurts","my disease","pooping","GI track","strokes","spots","sleep","ache","recovering","BLEEDING","reaction","Crohn","effect","affect", "symptom","feel","problem","fever","cramp","I was experiencing","I've been","I've had","rash","inflammation","bleeding","depression","anxiety","stool","Stool","depressed","having pain","abdominal pain","medicine") two = c("meds","prescription","drug","treatment","infusion","injection","Vaccine","taking","prescribe","prescription","refill","take the","tabs","daily","tablet","pill","vaccinate","miralax","Miralax","laxative","Antibiotic","antibiotic","steroids","supplement","My medication","my medication","vaccine","shot","flu shot","oral","Flu shot","the medication","Walgreens","walgreens","CVS","cvs","pharmacy","Pharmacy","over the counter","mg","miligrams","dose","dosage","pro biotic","probiotic","Probiotic","Probiotic","tylenol","Entyvio","entyvio","6MP","6mp","Asprin","asprin","Apriso","Allopurinol","Asacol","Azulfidine","azathioprine","Budesondie","Entocort","Canasa","antidepressants","Cimzia","Cipro","Creon","Colazal","Cortenema","Cortifoam","Dipentum","Entocort","Flagyl","humira","Humira","Imuran","immodium","Immodium","Lialda","methylprednisolon","Natalizumab","NyQuil","Ibuprofen","Pentasa","Prilosec","Prevacid","Aciphex","Protonix","Methotrexate","Nexium","Dexilant","Prednsione","Phenergan","Purinethol","Remicade","Rowasa","Simponi","Solu-Medrol","Prozac","Stelara","Tylenol","Useris","vicodin","Vicodin","Zosyn")  three = c("scheduling","apt","appointment","see me","see her","see him","see Dr","see the","seeing","appt","I can make","schedule","come in","be there","head over","followup","visit","SEE OR","meet")  four = c("lab","Lab","results","blood test","CBC","blood panel","draw","result","blood work","Quest","quest diagnostic","sample","drew blood","tests","CRP","test for","bloods","more blood","this test","my blood","Vitamin D","vitamin D","Vitamin d","iron","glucose") five = c("insurance","cost","careplan","expensive","money","health plan","\\$","hemoglobin","paystub","Blue Shield","financial","funds","PPO","HMO","Tricare","tricare","medical bills","pricing","Remistart","remistart","Co-Pay","co-pay","Healthcare") six = c("E-mail","email","@[gmail.com](http://gmail.com/)","[altour.com](http://altour.com/)","@[mednet.ucla.edu](http://mednet.ucla.edu/)","phone","number","my cell","fax","message","Email","error","call","get a hold of","contact","speak","mail","Zip code","located","location","address") seven = c("colonoscopy","procedure","scopy","MRI","PT scan","Petscan","CT","CAT","x-ray","X-ray","surgery","biopsy","biop","TB test","tuberculosis") eight = c("Patient has indicated there are changes to","Patient has indicated there are no changes","See attachment...") nine = c("Thank","thank","Hi","hi","Hey","hey","Hello","hello","Ok","ok","Yes","yes","thx","Testing","testing","Merry Christmas","Good Morning","Good morning","Good afternoon","Good Afternoon","good afternoon","Happy New Year","Happy Thanksgiving", "Nice","Happy")  cats = list(one,two,three,four,five,six,seven,eight,nine)  for(g in 1:length(cats)){  res = rep(0, nrow(MessagesPH))  for(i in 1:length(cats[[g]])){  res = res+as.numeric(grepl(cats[[g]][i], MessagesPH[,3]))  }  MessagesPH[which(res>0),2] = g + 10*MessagesPH[which(res>0),2] } for(j in 1:nrow(MessagesPH)){  if(MessagesPH[j,2] == 9){  if(grepl("\\?", MessagesPH[j,3])){  MessagesPH[j,2]=0  }  else{  if(length(strsplit(MessagesPH[j,3]," ")[[1]])>15){  MessagesPH[j,2]=0  }  }  } } for(y in 1:nrow(MessagesPH)){  if(grepl("New medication was added on", MessagesPH[y,3])){  MessagesPH[y,2]=8  } } for(n in 1:nrow(MessagesPH)){  if(grepl("<p>", MessagesPH[n,3])){  MessagesPH[n,2]=8  } } #Phone number searcher for(w in 1:nrow(MessagesPH)){  if(MessagesPH[w,2]==0){  Test= MessagesPH[w,3]  Test= as.numeric(strsplit(Test,"")[[1]])  count = 0  NAcount = 0  for(z in 1:length(Test)){  if(![is.na](http://is.na/)(Test[z])){  count = count + 1  NAcount = 0  if(count==10){  MessagesPH[w,2]= 6  break  }  }  else{  if(NAcount==2 && count > 0){  count = 0  NAcount = 0  }  if(NAcount<2 && count > 0){  NAcount = NAcount + 1  }  }  }  } } ######################################  ########THE CLEANER: Get rid of 9's and 8's######## remove = NULL for(w in 1:nrow(MessagesPH)){  if((MessagesPH[w,2]-9)%%10==0){  if(((MessagesPH[w,2]-9)/10)==0){  remove = c(remove,w)  }  MessagesPH[w,2] = (MessagesPH[w,2]-9)/10  }  if((MessagesPH[w,2]-8)%%10==0){  if(((MessagesPH[w,2]-8)/10)==0){  remove = c(remove,w)  }  } } MessagesPH = MessagesPH[-unique(remove),]  #############Use wisely################# write.csv(MessagesPH, "Categories2.csv") ########################################  #######Category Frequency Printer########### x = table(MessagesPH[,2]) for(h in 0:7){  print(h)  print(100*sum(x[which(grepl(as.character(h), rownames(x)))])/6193)  } #############################################  ######################HEATMAP######################### #1.create blank matrix Heat = matrix(0,nrow = 7, ncol = 7) #2. THE LOOP tab = table(MessagesPH[,2]) names = row.names(tab) for(x in 1:nrow(Heat)){  for(y in 1:ncol(Heat)){  for(z in 1:length(names)){  if((x %in% as.numeric(strsplit(names[z],"")[[1]])) && (y %in% as.numeric(strsplit(names[z],"")[[1]]))){  Heat[x,y] = Heat[x,y] + tab[z]  }  }  } }  diag(Heat) = tab[2:8] color = heat.colors(256) color = color[256:1] #heatmap(Heat, main ="Overlap of Categories in Pairs", Rowv=NA, Colv=NA, labRow = c("Medications","Symptoms","Appointments","Labs","Finance/Insurance","Communications","Procedures"),labCol = c("Medications","Symptoms","Appointments","Labs","Finance/Insurance","Communications","Procedures"), col = color, scale= "none", margins=c(5,10),symm=TRUE,revC=TRUE) heatmap.2(Heat, main ="Overlap of Categories in Pairs", Rowv=NA, Colv=NA, labRow = c("Medications","Symptoms","Appointments","Labs","Finance/Insurance","Communications","Procedures"),labCol = c("Medications","Symptoms","Appointments","Labs","Finance/Insurance","Communications","Procedures"), col = color, margins=c(5,10),symm=TRUE,revC=TRUE) ####Sample Test For Accuracy Creator#################### set.seed(100) rownumber = sort(sample(1:nrow(MessagesPH),size=100, replace=FALSE))  subset = MessagesPH[rownumber,3] subset = cbind(exam,subset)  write.csv(subset, "Catergoriestest.csv", row.names=FALSE)  subsetfull = table(MessagesPH[rownumber,2])  for(h in 0:7){  print(h)  print(100*sum(subsetfull[which(grepl(as.character(h), rownames(subsetfull)))])/100)   } ########################################################  ###############Accuracy Checker######################### Testresults = read.csv("Mastertest.csv",header = TRUE)  Computer = matrix(0,nrow = 100, ncol = 9) Dan = matrix(0,nrow = 100, ncol = 9) Aria = matrix(0,nrow = 100, ncol = 9) Courtney = matrix(0,nrow = 100, ncol = 9)  populate = function(frame,data){    for(u in 1:nrow(frame)){  for(g in 9:1){  if((data[u]-g)%%10 == 0){  frame[u,g] = 1  data[u] = (data[u]-g)/10  }   }    }  return(frame) }   Computer = populate(Computer, Testresults[,2])  Dan = populate(Dan, Testresults[,3])  Aria = populate(Aria, Testresults[,4])  Courtney = populate(Courtney, Testresults[,5])   scores = rep(0,300) underscore = rep(0,300) overscore = rep(0,300)  for(v in 1:nrow(Computer)){ scores[v] = sum(Computer[v,] != Dan[v,])  scores[v+100] = sum(Computer[v,] != Aria[v,]) scores[v+200] = sum(Computer[v,] != Courtney[v,]) }  for(o in 1:nrow(Computer)){  for(x in 1:9){  if(Computer[o,x]-Dan[o,x]<0){  underscore[o] = underscore[o]-1  }  if(Computer[o,x]-Aria[o,x]<0){  underscore[o+100] = underscore[o+100]-1  }  if(Computer[o,x]-Courtney[o,x]<0){  underscore[o+200] = underscore[o+200]-1  }  } }   for(o in 1:nrow(Computer)){  for(x in 1:9){  if(Computer[o,x]-Dan[o,x]>0){  overscore[o] = overscore[o]+1  }  if(Computer[o,x]-Aria[o,x]>0){  overscore[o+100] = overscore[o+100]+1  }  if(Computer[o,x]-Courtney[o,x]>0){  overscore[o+200] = overscore[o+200]+1  }  } } hist(scores,  main="Histogram for Raw Differences between Program and Doctor Categorization",  xlab ="Differences", border="blue", col="green", ylim=c(0,250)) hist(underscore,  main="Histogram for Underestimations of Categories by Program relative to Doctor",  xlab ="Number of Missed Categories", border="orange", col="red", ylim=c(0,250)) hist(overscore,  main="Histogram for Overestimations of Categories by Program relative to Doctor",  xlab ="Number of Missed Categories", border="brown", col="blue", ylim=c(0,250)) |
| --- |
